# Farmers' perception on the importance of variegated grasshopper (*Zonocerus variegatus *(L.)) in the agricultural production systems of the humid forest zone of Southern Cameroon

**DOI:** 10.1186/1746-4269-2-17

**Published:** 2006-03-30

**Authors:** Sévilor Kekeunou, Stephan Weise, Jean Messi, Manuel Tamò

**Affiliations:** 1 International Institute of Tropical Agriculture, Humid Forest Eco-regional Centre, Yaounde, Cameroon; 2 Zoology laboratory, Faculty of Science, University of Yaounde, Cameroon; 3 International Institute of Tropical Agriculture, Biological Control Center for Africa, Cotonou, Benin

## Abstract

**Background:**

*Zonocerus variegatus *(Linnaeus, 1758) (Orthoptera: Pyrgomorphidae) is known as an agricultural pest in West and Central Africa. However, its importance in the agricultural production system in Cameroon has not been investigated. The study assesses farmers' perception on the importance of *Z. variegatus *in the agricultural production systems of the humid forest zone of Southern Cameroon.

**Methods:**

Research was carried out in 5 villages of each of three Agro-Ecological, Cultural and Demographic Blocks (AECD-Blocks) of the Forest Margin Benchmark Area (FMBA). In each village, a semi-structured survey was used; male and female groups of farmers were interviewed separately.

**Results:**

*Z. variegatus *is present throughout the humid forest zone of Southern Cameroon, where it is ranked as the third most economically important insect pest of agriculture. In the farmers' opinion, *Z. variegatus *is a polyphagous insect with little impact on young perennial crops. The length of the pre-farming fallow does not affect *Z. variegatus *pest pressure in the following crops. The increased impact of the grasshopper observed today in the fields, compared to what existed 10 years ago is as a result of deforestation and increase in surface of herbaceous fallow. The damage caused by *Z. variegatu*s is higher in fields adjacent to *C. odorata *and herbaceous fallows than in those adjacent to forests and shrubby fallows. The fight against this grasshopper is often done through physical methods carried out by hand, for human consumption. The farmers highlight low usage of the chemical methods and a total absence of biological and ecological methods.

**Conclusion:**

Farmers' perception have contributed to understanding the status of *Z. variegatus *in the humid forest zone of Southern Cameroon. The results are in general similar to those obtained in other countries.

## Background

In general, from the human point of view, any insect that is not at the right place is a pest (Williams, cited by [1]). Pests are also harmful or awkward species, which need to be controlled for economic or social reasons (Clark, cited by [1]). *Zonocerus variegatus *is reported in the literature as a pest of many crops in West and Central Africa. The first reports on damages of this grasshopper on crops were in 1910 by Peacock and Lamborns in Southern Nigeria, Schoutedem and Mayne in Zaire and Small in Uganda (*In *[2]). Its geographical range and impact on the crops increase with time. In 1948, *Z. variegatus *accounted for 10% yield loss in the banana harvest in Guinea [2]. On garden eggs, it can cause 25 – 80% yield loss [2]. In Nigeria, it causes 50% loss in annual cassava yield [4]. *Z. variegatus *has been implicated in the transmission of okra mosaics viruses in Ivory Coast and cowpea mosaics viruses in Nigeria [3,5]. It is thought to be responsible for the transmission of the bacterial burn of cassava in Nigeria [2,3]. Due to the high damages it inflicts on crops, it is considered as an important agricultural pest in Nigeria [4,6-8], Ivory Coast [6], Ghana [4], Congo Brazzaville [9], Southern Benin (Coffi, cited by [4]) and in the Sahel [10]. *Z. variegatus *is currently the most important of grasshopper pests for crops in the humid forests of low altitude and savannas of West and Central Africa [2,4,11,12]. However, no scientific literature exists on the pest status of *Z. variegatus *in Cameroon. Apart from two works [13,14] and one report [5], most other literature on the problems caused by *Z. variegatus *in Central and West Africa only have generalities on Cameroon [4-6,9,15,16]. Determining the importance of an insect in any agro-ecosystems, contributes to the description of its status [17]. Such information is necessary for the formulation of a good pest management strategy.

This paper present the results of a group survey, administratered to the farmers of the humid forest zone of Southern Cameroon, to assess the importance of variegated grasshoppers *Zonocerus variegatus *in the agricultural production systems. The survey approach was justified by the concern of having a fast idea on the *Z. variegatus *status in the production systems in the southern Cameroon and the need of collaboration between farmers, scientists, and extension services in term of priorities definition about the crop protection strategies. Recent trends in agricultural research and development emphasize the need for farmer participation [18]. There has been increasing interest in the incorporation of farmer's knowledge into agricultural research and development programs [19]. The Participatory Action Research framework provides one useful approach towards achieving farmers' capacity building [18]. Farmers in general are good decision-markers (Goldman, cited by [20]) and their views have contributed to the understanding of various aspects of the bio-ecology of insects and the real situation of other pests. Taking their knowledge base and combining them with scientists'/extentionists' expertise can contribute to the improvement of local practices in pest management [18]. An ethnoentomological study, conducted in Tharu, a village in Nepal, offered a basis to improve pest management programs in terms of efficacy and acceptance [18]. In African farmer communities, insects are much more known as pest though others such as *Apis mellifera *(Hymenoptera: Apidae), *Gryllotalpa africana *(Orthoptera: Gryllotalpidae), *Gryllus bimaculatus *(Orthoptera: Gryllidae) have a great importance in the communities. In general, *A. mellifera *(bees) produce honey. In West Cameroon *G. africana *are heralding misfortune whereas the cries of *G. bimaculatus *at certain periods of the year, announce good news (Kwecheu Marie, Personal Communication). Farmers' knowledge of insects varies in quality and quantity depending on their interest in the subject, their environment, and the relevance of insects to their lives [18].

The objective of the present study was to determine farmers' perception on: (1) the incidence of *Zonocerus variegatus *in the humid forest zone of Southern Cameroon; (2) the factors responsible for variations of *Z. variegatus *incidence in the humid forest zone of Southern Cameroon; and (3) to assess how the farmers manage this grasshopper in their agro-ecosystems.

## Materials and methods

### Study site

**Figure 1:**
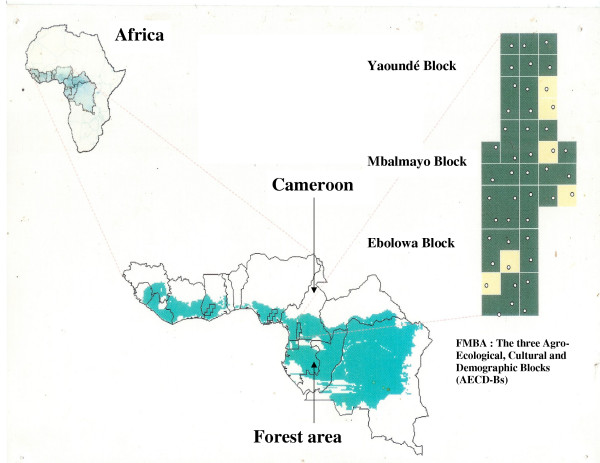
The Forest Margin Benchmark Area (FMBA) of the humid forest zone of the Southern-Cameroon.

The work was carried out in 15 villages of the humid forest zone of Southern Cameroon (3°27'-4°10'N and 11°32'-11°49'E) (Figure 1). These villages were selected among the 45 Forest Margin Benchmark Area (FMBA: 1.54 millions hectares) reference villages. Serving as a focal point for strategic diagnostic research in the sub-region, the benchmark approach was developed and implemented through the Eco-regional Program for the Humid and Sub-Humid Tropics of Sub-Saharan Africa (EPHTA). The 45 villages are situated along a gradient of natural resource utilization intensification, represented by 3 agro-ecological, cultural and demographic blocks (AECD-Blocks) namely Yaounde, Mbalmayo and Ebolowa (Figure 1). Five out of 15 villages of each block were selected for the study. These were Nkongmesse, Nkolmelok, Nkometou-II, Akak-II and Etoud for the Yaounde block; Awae, Evindissi, Ngat, Mvoutessi and Nkolmetet for the Mbalmayo block and Minsélé, Mekoe, Mengomo, Akok and Obang-II for the Ebolowa blocks. The 3 AECD-Blocks differ in several aspects. Yaounde has a high human density (14–88 habitant/km^2^); whereas low human population densities are observed in Ebolowa (2–15 habitant/km^2^) and intermediates (10–48 habitant/km^2^) in Mbalmayo [21]. On the agro-ecological level, forest degradation is more pronounced around Yaounde than in the Ebolowa, which still has some pockets of primary forest [21]. Mbalmayo constitutes an intermediate block. Farmers in these areas all practice slash and burn agriculture [22]. In the Yaounde block, the length of fallow is about three years shorter than that of Ebolowa (7.5 years) while that of Mbalmayo block is a transition (5.4 years). The main food crops are banana, plantain, cocoyam, cassava, yam and groundnut [22]. Cucurbits, okra, vegetables and spices are secondary crops [22] while cocoa and coffee are the main cash crops of this area. Their production is mainly in small-scale, through domestic or family exploitation. These areas have an equatorial climate type with two dry seasons and two rainy seasons. The average annual rainfall is 1510 mm, 1643 mm and 1820 mm in Yaounde, Mbalmayo and Ebolowa respectively. From an ethnological viewpoint, the "Etons", "Ewondoses" and the "Bulus" are the main ethnic groups in the Yaounde, Mbalmayo and Ebolowa blocks respectively. With regard to the diet, the Bulus consume more sauces made based on almond of the fruits of *Irvingia gabonensis *(named 'Ndo'o') while Etons and Ewondoses consumes more 'Okok' (pasta product made containing leaves of *Gnetum africanum*) and 'Kwem' (pasta product made containing leaves of *Manihot esculenta*) respectively (Lema Ngono, Personal Communication).

### Survey

Information was obtained in each village between August and October 1998 using the rapid rural appraisal methods (RRAM) [23-25]. There were 17 questions, divided in three parts; 5 relating to the annual crops and, 5 others relating to the young perennial crops and 7 general questions. The final questionnaire was prepared based on a preliminary one that was tested in Bikok and Abang, two other villages different from those used in our investigations. Based on the results of this pre-test, we decided to work with men and women farmers' groups separately in each village. We selected farmers growing annual or perennials crops. A total of 30 groups (389 farmers: 164 women and 225 men) were interviewed. Each group had 8 to 21 farmers aged 18 to 50 years. Participants were recruited in 3 strata based on the 3 blocks of the 'Benchmark' [23,24]. Interviews of two hours per group were conducted at different hours of the same day for the two groups. Farmers were asked to rank the importance of *Zonocerus variegatus *among the other insect pest of the humid forest zone of the Southern Cameroon and rate the impact of *Z. variegatus *on crops in the humid forest zone of Southern Cameroon. Farmers were also asked to state factors, which influence the pest status of *Z. variegatus*, the methods used against the pest and to state what they need to suppress the pest population. In the questionnaire, we used opened-ended and close-ended questions.

Questions were asked in the local language and/or French. During the survey in each village, a sample of *Zonocerus variegatus *was shown to the farmers to enable them recognize the insect. For the various rankings, scores (described *In *[25]) were used while the farmers answered the other questions directly.

### Statistical analysis

Data were analyzed by the Kruskal-Wallis test using the 'Nonparametric One Way' ('NPAR1WAY WILCOXON ') procedure of the software SAS ('Statistical Analysis Systems' version 8). All probabilities were appreciated at the 5% confidence level.

## Results

### Incidence of *Zonocerus variegatus *in the humid forest zone of Southern Cameroon

*Zonocerus variegatus *is present in all the surveyed villages. In all these villages, variegated grasshopper is well known to the farmers and they could describe the appearance of both nymphs and adults. All the farmers' group rate it as an insect pest. *Z. variegatus *is ranked the 3^rd ^most important insect pest in the area and together with borers and scale insects cause about 54% of the insect pest problems on annual crops (Table 1). On young perennial crops, mirid, borers and caterpillars are the major constraints and they cause about 59% of insect pest problems while *Z. variegatus *is a minor pest (Table 1). However, for the females groups of Yaoundé (for the annual and perennial crops) and Mbalmayo (for the perennial crops only), these pests have equal importance (Table 1).

**Table 1:** Relative importance (%) of the main insect pests on annual and perennial crops in the humid forest zone of Southern – Cameroon.

**All**										
**Crops**	**AECD-Blocks**	**Termites**	**Borers**	**Caterpillars **(Without Borers)	**Aphids**	**Mirids**	**Scales Insects**	**Variegated grasshopper**	**Bugs **(Without Mirids)	**p-value**

Annual crops	Yaounde	9	16	9	10	6	19	16	14	<0.001
	Mbalmayo	2	21	10	9	7	22	16	13	<0.0001
	Ebolowa	4	22	12	13	5	17	14	15	<0.0001
	p-value	<0.05	NS	NS	NS	NS	NS	NS	NS	
	Mean	5	20	10	11	6	20	15	14	<0.0001

Young Perennial Crops	Yaoundé	14	14	12	11	21	18	7	5	<0.01
	Mbalmayo	1	19	19	10	28	13	8	2	<0.0001
	Ebolowa	3	22	14	14	28	5	11	4	<0.0001
	p-value	<0.001	NS	NS	NS	NS	<0.05	NS	NS	
	Mean	6	18	15	11	25	12	9	4	<0.0001

**Female**										

Annual crops	Yaounde	11	16	9	13	9	19	12	14	NS
	Mbalmayo	3	21	10	12	9	22	12	13	0.0386
	Ebolowa	5	22	11	14	3	17	14	16	0.0236
	p-value	NS	NS	NS	NS	NS	NS	NS	NS	
	Mean	6	19	10	13	7	19	12	14	<0.0001

Young Perennial Crops	Yaoundé	14	15	7	14	18	23	3	11	NS
	Mbalmayo	2	21	19	10	29	14	2	5	0.0005
	Ebolowa	4	21	15	15	22	7	13	4	NS
	p-value	NS	NS	NS	NS	NS	NS	NS	NS	
	Mean	7	19	14	13	23	15	6	6	<0.0001

**Male**										

Annual crops	Yaounde	7	17	10	8	3	20	21	15	0.0072
	Mbalmayo	2	21	10	7	6	23	20	13	0.0003
	Ebolowa	3	23	13	12	7	17	14	14	0.0002
	p-value	NS	NS	NS	NS	NS	NS	NS	NS	
	Mean	4	20	11	9	5	20	18	14	<0.0001

Young Perennial Crops	Yaoundé	14	13	16	8	24	13	11	3	0.0404
	Mbalmayo	0	18	19	10	27	13	12	2	0.0007
	Ebolowa	3	24	13	12	33	2	10	4	0.0006
	p-value	0.008	NS	NS	NS	NS	NS	NS	NS	
	Mean	6	18	16	10	28	9	11	3	<0.0001

The values are averages of the scores for 10 groups (5 male and 5 female groups) of farmers interviewed per block; p-value is the significance levels of a non parametric test of Kruskal-Wallis; NS = no significant (p > 0.05). AECD-Blocks = Agro-Ecological, Cultural and Demographic Blocks.

**Table 2:** Food sources of *Zonocerus variegatus *among the annual and perennial crops in the humid forest of Southern Cameroon.

**All**						
**Crops**	**AECD-Blocks**	**General feeder (Polyphagous) **	**Oligophagous**	**Monophagous**	**No crop attacked**	**Absent in the village**

Annual crops	Yaounde	60	40	0	0	0
	Mbalmayo	70	30	0	0	0
	Ebolowa	70	30	0	0	0
	Mean	67	33	0	0	0

Young Perennial Crop	Yaounde	20	20	10	50	0
	Mbalmayo	20	20	10	50	0
	Ebolowa	30	50	0	20	0
	Mean	23	30	7	40	0

**Female**						

Annual crops	Yaounde	60	40	0	0	0
	Mbalmayo	60	40	0	0	0
	Ebolowa	60	40	0	0	0
	Mean	60	40	0	0	0

Young Perennial Crop	Yaounde	20	0	0	80	0
	Mbalmayo	40	0	0	60	0
	Ebolowa	40	40	0	20	0
	Mean	33	13	0	53	0

**Male**						

Annual crops	Yaounde	60	40	0	0	0
	Mbalmayo	80	20	0	0	0
	Ebolowa	80	20	0	0	0
	Mean	73	27	0	0	0

Young Perennial Crop	Yaounde	20	40	20	20	0
	Mbalmayo	0	40	20	40	0
	Ebolowa	20	60	0	40	0
	Mean	13	47	13	33	0

The values are averages of the scores for 10 groups of farmers (5 male and 5 female groups) interviewed per block; AECD-Blocks = Agro-Ecological, Cultural and Demographic Blocks

Most farmers' groups (67%) reported that *Zonocerus variegatus *is a general feeder (polyphagous insect) (Table 2) that attacks all the annual crop species. These perceptions were higher at male (73% of groups) than female (60% of groups) (Table 2). Its food host range included cassava, groundnut and vegetables. None of the farmers' groups rated *Z. variegatus *as a monophagous pest on annual crops (Table 2). On young perennial crops, most groups (40%) perceived *Z. variegatus *as a non-harmful insect (Table 2). This perception was more pronounced in female groups (53%) than male groups (33%) (Table 2). Few farmers (23% of groups), especially the female (33% of groups) reported *Z. variegatus *as being olygophagous (Table 2) compared to only 13% of men groups. Their food range on perennial crops was more restricted to pear, cocoa, coffee, orange, oil palm and plum. *Z. variegatus *was rated by 7% (female only) of the groups as being monophagous (Table 2). In this monophagous behaviour, the grasshopper appeared to be a specialist feeder on pear or palm tree.

### Factors responsible for variations in *Zonocerus variegatus *incidence in the humid forest zone of Southern Cameroon

#### Factors mentioned by the farmers

Compared to the scenario 10 years ago, most of farmers' groups (84%) surveyed in the humid forest zone reported that *Zonocerus variegatus *pressure in the fields has increased. These perceptions were higher in male (87% of groups) than in female groups (80% of groups). This increase of *Z. variegatus *incidence has been significant (73% of groups; 87% of male and 60% of female) mainly because of the increase in the surface of herbaceous fallow (73% of groups) and deforestation (60% of groups) (Table 3). Males (53% of groups) also give more importance to some factors such as high reproductive rate of *Z. variegatus*. Females perceived these factors as minors (Table 3). Most of other factors presented in table 3 were minors. Some factors like lack of human consumption, wide colonization of the fields by weeds, presence of cassava were only reported by males, while possible change in the *Z. variegatus *species has been proposed by females (Table 3).

**Table 3:** Reasons for the increase incidence of *Zonocerus variegatus *in crop fields in the humid forest zone of Southern Cameroon.

	**All**				**Female**				**Male**			
**Reasons **	**Ydé**	**Mbyo**	**Ebwa**	Means	**Ydé**	**Mbyo**	**Ebwa**	Means	**Ydé**	**Mbyo**	**Ebwa**	Means

Increased surfaces of herbaceous and *C. odorata *fallows	50	90	80	73	40	80	80	67	60	100	80	80
Deforestation	60	60	60	60	40	60	60	53	80	60	60	67
High reproductive rate of *Z. variegatus*	20	80	20	40	0	80	0	27	40	80	40	53
Lack, suppression or reduction of control measures against *Z. variegatus*	30	20	20	23	20	0	0	7	40	40	40	40
Increased food host range of *Z. variegatus*	40	20	10	23	60	20	0	27	20	20	20	20
Absence, suppression or reduction of human consumption of *Z. variegatus*	10	10	10	10	0	0	0	0	20	20	20	20
Wide colonization of the fields by weeds	10	0	10	7	0	0	0	0	20	0	20	13
Increase sun shine levels	10	20	10	13	0	20	20	13	20	20	0	7
Favorable climatic conditions	0	20	30	17	0	20	40	20	0	20	20	13
Change of the crop types	10	0	0	3	20	0	0	7	20	0	0	7
Reduction of spaces in sugar cane crop	10	0	10	7	20	0	0	7	0	0	20	7
Climatic changes	20	20	30	23	0	20	20	13	40	20	20	27
Presence of cassava	0	10	0	3	0	0	0	0	0	20	0	7
Possible change of the species of *Z. variegatus*	10	0	0	3	20	0	0	7	0	0	0	0

The values (% of answer) are averages for 10 groups of farmers (5 male and 5 female groups) interviewed per block. AECD-Blocks = Agro-Ecological, Cultural and Demographic Blocks. Ydé : Yaoundé, Mbyo: Mbalmayo, Ebwa: Ebolowa.

#### Factors presented to the farmers

##### - variations in Zonocerus variegatus incidence according to fields types

All field types were damaged by *Zonocerus variegatus *(Table 4) but, 51% of the damage was associated to food crop fields ('Affub bidi' and 'Affub owondo'). *Z. variegatus *pressure was low in dry season fields ('Esseps'); young perennial crops fields; garden crops and marsh' fields ('Assans'). The pressure of *Z. variegatus *in groundnut fields ('affub owondo') is higher in the Mbalmayo block. In Assans and 'Garden crops', this pressure is significantly higher in the Yaounde and Mbalmayo blocks (Table 4). However, for the female groups, these block effects appeared significant only in the garden crops (Table 4).

**Table 4:** Relative importance (%) of *Zonocerus variegatus *pressure in different fields types in the humid forest zone of Southern Cameroon.

**All**							
**AECD-Blocks**	**Affub owondo**	**Affub bidi**	**Assan**	**Essep**	**Garden crops**	**Young perennial crop**	**p-value**

Yaounde	10	34	21	9	20	5	<0.0001
Mbalmayo	31	28	9	5	17	9	<0.0001
Ebolowa	19	28	20	12	7	14	<0.001
p-value	<0.01	NS	<0.05	NS	<0.05	NS	
Means	20	30	17	9	15	9	<0.0001
**Female**							
Yaounde	11	38	21	9	17	3	0.0121
Mbalmayo	29	21	12	8	25	5	0.0021
Ebolowa	20	28	22	9	5	16	0.0346
p-value	NS	NS	NS	NS	0.0380	NS	
Means	20	29	18	9	16	8	0.0001
**Male**							
Yaounde	10	31	21	9	22	7	0.0029
Mbalmayo	33	36	6	3	10	12	0.0010
Ebolowa	19	28	19	15	9	11	0.0175
p-value	0.0209	NS	0.0220	NS	0.0422	NS	
Means	21	32	15	9	14	10	<0.0001

The values are averages of the scores for 10 groups of farmers (5 male and 5 female groups) interviewed per block. Affub owondo is Grandnut' field. Essep is dry season' fields. Assan is marsh' fields. p-value is the significance levels of a non parametric test of Kruskal-Wallis; NS = nonsignificant (p > 0.05). AECD-Blocks = Agro-Ecological, Cultural and Demographic Blocks.

##### - variations in Zonocerus variegatus incidence according to the age of the pre-farming fallow

The length of pre-farming fallow did not influence the extent of *Zonocerus variegatus *pressure in field crops (93% of groups) (Table 5). In the male and female groups, there were no significant differences in *Z. variegatus *pressure in the 3 types of fallow (Table 5). Similarly, the magnitude of the pressure did not differ significantly between blocks (Table 5).

**Table 5:** Influence of age of the pre-farming fallow on the pressure (%) of *Zonocerus variegatus*in crop fields established at the same site.

**All**				
**AECD-Blocks**	**Fallow of 1 -5 years**	**Fallow of 5 -10 years**	**Fallow > 10 years**	p-value

Yaounde	39	31	30	NS
Mbalmayo	40	30	30	NS
Ebolowa	33	33	33	NS
p-value	NS	NS	NS	
Means	37	32	31	NS
**Female**				
Yaounde	33	33	33	NS
Mbalmayo	47	27	27	NS
Ebolowa	33	33	33	NS
p-value	NS	NS	NS	
Means	38	31	31	NS
**Male**				
Yaounde	44	29	27	NS
Mbalmayo	33	33	33	NS
Ebolowa	33	33	33	NS
p-value	NS	NS	NS	
Means	37	32	31	NS

The values are averages of the scores for 10 groups of farmers (5 male and 5 female groups) interviewed per AECD-Blocks; p-value is the significance levels of a non parametric test of Kruskal-Wallis ; NS = nonsignificant (p > 0.05); AECD-Blocks = Agro-Ecological, Cultural and Demographic Blocks.

##### - variations in Zonocerus variegatus incidence according to the type of adjacent fallow

The magnitude of *Zonocerus variegatus *pressure varied with the type of adjacent fallow in field crops (93% of male and 87% of female). In the male and female groups, it is higher in fields adjacent to *Chomolaena odorata *fallow and herbaceous fallow than in forest or shrub fallows (Table 6).

**Table 6:** Influence of fallow types on *Zonocerus variegatus *pressure (%) in adjacent crop fields in the humid forest zones of Southern Cameroon

**All**					
**AECD-Blocks**	***C.odorata*fallow**	**Herbaceous fallows**	**Shrubs fallow**	**Forest**	p-value

Yaounde	52	35	10	5	<0.0001
Mbalmayo	44	40	11	7	<0.0001
Ebolowa	51	39	9	2	<0.0001
p-value	NS	NS	NS	NS	
Means	49	38	10	4	<0.0001
**Female**					
Yaounde	54	29	10	7	0.0052
Mbalmayo	45	40	10	5	0.0024
Ebolowa	50	43	7	0	0.0007
p-value	NS	NS	NS	NS	
Means	50	37	9	40	<0.0001
**Male**					
Yaounde	49	40	9	2	0.0007
Mbalmayo	42	39	11	8	0.0037
Ebolowa	51	34	11	4	0.0010
p-value	NS	NS	NS	NS	
Means	47	38	10	5	<0.0001

The values are averages of the scores for 10 groups of farmers (5 male and 5 female groups) interviewed per AECD-Blocks; p-value is the significance levels of a non parametric test of Kruskal-Wallis NS = nonsignificant (p > 0.05); AECD-Blocks = Agro-Ecological, Cultural and Demographic Blocks.

##### - variations in Zonocerus variegatus incidence according to type of season

The pressure of *Zonocerus variegatus *on annual and perennial crops is higher in the short dry season and highest in the great dry season. *Z. variegatus *damage is very low in the fields during the rainy season. No significant difference was observed between the fields (Table 7). However, on the perennial crops, differences (non-significant) in *Z. variegatus *incidence variations (according the seasons) have been reported by females of Ebolowa and Mbalmayo blocks. (Table 7). According to farmers, there was a block effect in the dry season on young perennial crops with the Ebolowa block having the highest damage (Table 7). However, there was no block effect in gender perceptions in the annual and perennials crops (Table 7).

**Table 7:** Influence of season on the population pressure (%) of *Zonocerus variegatus *on annual and perennial crops in the humidforest zones of Southern Cameroon.

**Crops**	**AECD-Blocks**	**Long dry season **	**Short rainy season **	**Short dry season **	**Long rainy season **	p-value
Annual Crop	Yaoundé	72	4	16	9	<0.0001
	Mbalmayo	70	7	11	13	<0.0001
	Ebolowa	64	10	27	0	<0.0001
	p-value	NS	NS	NS	NS	
	Means	69	7	18	7	<0.0001

Young Perennial Crops	Yaoundé	25	3	12	1	<0.05
	Mbalmayo	36	5	10	10	<0.05
	Ebolowa	63	9	29	0	<0.0001
	p-value	<0.05	NS	<0.05	NS	
	Means	41	5	17	4	<0.0001

**Female**						

Annual Crop	Yaoundé	76	1	13	10	0.0054
	Mbalmayo	68	5	12	15	0.0071
	Ebolowa	67	9	24	0	0.0015
	p-value	NS	NS	NS	NS	
	Means	69	5	16	8	<0.0001

Young Perennial Crops	Yaoundé	15	0	5	0	NS
	Mbalmayo	31	6	13	10	NS
	Ebolowa	69	4	27	0	0.0017
	p-value	NS	NS	NS	NS	
	Means	38	3	15	3	0.0014

**Male**						

Annual Crop	Yaoundé	68	6	19	7	0.0061
	Mbalmayo	72	9	9	10	0.0080
	Ebolowa	61	10	29	0	0.0015
	p-value	NS	NS	NS	NS	
	Means	67	8	19	6	<0.0001

Young Perennial Crops	Yaoundé	34	6	18	2	NS
	Mbalmayo	40	3	7	9	NS
	Ebolowa	57	13	30	0	0.0017
	p-value	NS	NS	NS	NS	
	Means	44	7	18	4	0.0002

The values are averages of the scores for 10 groups of farmers (5 male and 5 female groups) interviewed per AECD-Blocks; p-value is the significance levels of a non parametric test of Kruskal-Wallis; NS = nonsignificant (p > 0.05); AECD-Blocks = Agro-Ecological, Cultural and Demographic Blocks.

### Entomophagy

In the humid forest zone of southern Cameroon, only the adult stages of *Zonocerus variegatus *were an item of diet (63% of groups). This feeding behavior appeared more in Mbalmayo (90% of groups) and Yaoundé (70% of groups) blocks. *Z. variegatus *was eaten a little in Ebolowa block (30% of groups). Some people do not eat *Z. variegatus *because of its body odour (3% of groups) and the fact that, consumption would result in muscular and articular paralysis (6% of groups).

### Protection of crops against *Zonocerus variegatus*

To protect crops against *Zonocerus variegatus*, farmers of the humid forest zone of Southern Cameroon use chemical and physical methods. No biological or ecological methods were used (Table 8). The physical fight is very much used (77% of groups) (Table 8). Through this method, grasshoppers are collected mostly for human consumption as mentioned by 63% of the groups (61% of female and 60% of male) (Table 8). Very few groups (13%) collect *Z. variegatus *for use as baits in fishing. Chemical control of *Z. variegatus *populations was poorly practiced (27% of farmers) (Table 8). The insecticides used are décis^®^, diméthoate^®^, orthene^®^, cipercal^®^, furadan^® ^and methyl^®^. There were differences in the insecticide use in various fields. In Yaounde décis^® ^and furadan^® ^were not used while in the Mbalmayo block diméthoate^®^, orthene^®^, cipercal^® ^and methyl^® ^are not used. Insecticides are not used against *Z. variegatus *in Ebolowa. Rains are the only natural factors against *Z. variegatus *though its effect is only slightly perceived in the Yaounde and Mbalmayo blocks (Table 8). Some farmers groups (23%) do not have any means of fighting against *Z. variegatus *especially in the Ebolowa block (Table 8).

**Table 8:** Different methods used by farmers in the humid forest of Southern Cameroon of control *Zonocerus variegatus*.

**All**				
**AECD-Blocks**	**No method**	**Chemical method**	**Natural method (rain)**	**Physical method**

Yaounde	10	50	40	90
Mbalmayo	0	30	30	100
Ebolowa	60	0	10	40
Means	23	27	27	77
**Female**				
Yaounde	20	40	20	80
Mbalmayo	0	20	20	100
Ebolowa	60	0	0	40
Means	27	20	13	73
**Male**				
Yaounde	0	60	60	100
Mbalmayo	0	40	40	100
Ebolowa	60	0	20	40
Means	20	33	40	80

The values (%) are averages for 10 groups of farmers (5 male and 5 female groups) interviewed per AECD-blocks; AECD-Blocks = Agro-Ecological, Cultural and Demographic Blocks.

## Discussion

The study shows that farmers regard *Zonocerus variegatus *as an important annual crop pest in the humid forest zone of Southern Cameroon. It is a minor pest of young perennial crops. Similar results have also been reported in many countries in Africa, notably Nigeria [4,6-8], Ivory Coast [6], Ghana [4], Congo Brazzaville [9], Southern Benin (Coffi, cited by [4]) and the Sahel [10]. In most of these countries, *Z. variegatus *is known mainly as a food crop pest.

From the farmers' point of view, the definition of an insect pest has primarily economic implications, given that the insect causes significant damage to warrant the implementation of a control measure. This implies that having reliable information on crop damage at different pest densities is vital in understanding the interaction between pest and host plants. However, crop damage by *Zonocerus variegatus *is very difficult to evaluate, especially on food crops [2]. This underscores the importance of farmers' perceptions in understanding the pest status of a given insect [17]. Farmers' perceptions have contributed to the understanding of various aspects of the bio-ecology of insects [18]. These perceptions can be significantly different from scientific knowledge but have major implications for development [27,5,31,18].

The crops mentioned by the farmers in this study as food sources for *Zonocerus variegatus *have also been reported in previous studies [2,38]. For the farmers of southern Cameroon, groundnut, cassava and vegetables are the most susceptible crops to *Z. variegatus*. The majority of the authors are unanimous on the high vulnerability of cassava [4,6,7] while the susceptibility level of vegetables and groundnut varies from one country to another. Cassava, groundnut and vegetables are the principal food crops of the humid forest zone of Southern Cameroon [22]. It thus gets clear a need for developing strategies of protection of these crops against *Z. variegatus*.

Many reports have also mentioned the growing importance of *Zonocerus variegatus *as a pest elsewhere as in the humid forest zone of Southern Cameroon. For example, before 1950, *Z. variegatus *was already known as a pest of primary or secondary importance in most countries of intertropical Africa [2,5]. However, it was later reported as an important pest in the 1970s in Nigeria [6,7,12].

The farmers in this study recognize as Kumar [1] that several factors affect the pest behavior of *Zonocerus variegatus*. For example *Z. variegatus *avoids pure forest compared to deforested areas [6,3,32,2,4]. This preference of deforested areas had also been reported in earlier studies [6,7,2,37]. Deforestation induces biotopes and conditions favorable for *Z. variegatus *such as increase in surfaces of herbaceous fallow. In addition, after deforestation, empty spaces are occupied by *C. odorata*, the main host plant of *Z. variegatus*. These may partly explain the high pressure of *Z. variegatus *observed by the farmers in the fields adjacent to *C. odorata *fallow [8] and herbaceous fallow compared to fields adjacent to forests. The increased damage observed in the dry season suggests that it is a seasonal pest as reported in other studies [3]. Probably, rains (natural mortality factor) induce morbid fungical infections in the natural populations of *Z. variegatus *among other mortality factors that would reduce the pest population.

The study showed that, *Zonocerus variegatus *is an item of diet. This agrees with the findings of Iduwu & Modder [8] and Page & Richards [38] in Nigeria. In Nigeria as in Cameroon, the insect is eaten after being fried [8,38]. In fact, *Z. variegatus *is an important source of proteins [39].

In the present study, the main control method of *Zonocerus variegatus *in the humid forest of Southern Cameroon was through the physical removal of insects feeding on crops for human consumption. The consumption of *Z. variegatus *as control method has also been reported by Nigerian farmers [8]. However, physical control of *Z. variegatus *is generally difficult and not very effective. The high population of *Z. variegatus *in the humid forests of Southern Cameroon [33] indicates the lack of an adequate crop protection measure against the pest. Egg-pod exposure used by few Nigerian subsistence farmers is not known in southern Cameroon [4]. Southern Cameroon farmers seldom use chemical control, certainly because of the fall of the state aid, related to the economic crisis. This method, very much used by the farmers in Nigeria [8], was the main crop protection strategy in the tropical forest zones. Their utilization, in large areas has been one of the first important factors of reduction in crop loss due to pests [9]. However, because of their harmful effect on the environment, their utilization became weakly recommended [34,10,1,36]. The fact that biological control methods are not used here show that the 'green muscle' (biological acridicid) used successfully in the savannah zones may not have been introduced to farmers of Southern Cameroon. These farmers' perceptions showed a need to carry out an urgent control strategy in the agricultural production system against *Zonocerus variegatus*. However, the strategy needs to be directed a little more towards the integrated pest management as described by Modder [7]. An integration of the farmers is necessary [38].

This study also showed some perceptional gender differences in the humid forest zone of southern Cameroon. In Yaoundé (for the annual and perennial crops) and Mbalmayo (for the perennial crops only), females perceived the pest's incidence with an equal importance while males recognized *Z. variegatus *as one of the major and minor pests in the annual and perennial crops respectively. On young perennial crops, only the females rated *Z. variegatus *as monophagous. In these perennial crops, more female groups perceived *Z. variegatus *as non-harmful insect than male groups. The increase of *Z. variegatus *incidence in the fields has been more reported by men than women groups. For the female groups, block effect appeared significant only in the garden crops while in the male groups they also appeared in groundnut fields ('affub owondo') and Assan (Marsh field). More male groups observed that, the magnitude of *Zonocerus variegatus *pressure varied with the type of adjacent fallows in field crops. These perceptional gender differences have also been observed in Nepal [18]. In Nepal, with regard to the depredatory insects, men generally used more vague attributes like harmful or harmless, while women were more specific, often referring to the host plant [18]. The rate for instance, was referred to by women as a hole-making storage pest, while men did not refer to burrows at all [18]. This clearly reflected that in Nepal, women normally repair and clean the damage [18]. In fact, the perceptional gender differences have their origin in the division of labor [18]. In the humid forest zone of southern Cameroon, men and women spend most of their time in the field (agriculturals activities) but, women are also involved in tasks at household while other male tasks are community oriented.

## Conclusion

Farmers' perceptions have contributed to the understanding of the pest status of *Zonocerus variegatus *in the humid forest zone of Southern Cameroon. Farmers rate *Z. variegatus *as an important polyphagous pest on food crops that warrants urgent management or control. The pest incidence has increased because of deforestation and increase of herbaceous fallows. It is therefore necessary to also consider this grasshopper among the major pests in the national strategies of plant protection. Integrated Pest Management (IPM) strategy is a comprehensive approach that combines all rational strategies to reduce pest densities to tolerable levels while maintaining a safe quality environment [7]. These farmers' perceptions are in general similar to the results of most experimental studies but may stimulate researchers to identify new research areas. Farmers' perception can differ profoundly from scientific knowledge, having significant implications for development [18]. Both farmers and scientific knowledge have strengths and weaknesses [18].
